# Association between opioid analgesic therapy and initiation of buprenorphine management: An analysis of prescription drug monitoring program data

**DOI:** 10.1371/journal.pone.0227350

**Published:** 2020-01-10

**Authors:** Apostolos A. Alexandridis, Nabarun Dasgupta, Christopher L. Ringwalt, Wayne D. Rosamond, Paul R. Chelminski, Stephen W. Marshall

**Affiliations:** 1 Injury Prevention Research Center, University of North Carolina at Chapel Hill, Chapel Hill, North Carolina, United States of America; 2 Department of Epidemiology, Gillings School of Global Public Health, University of North Carolina at Chapel Hill, Chapel Hill, North Carolina, United States of America; 3 Department of Medicine, School of Medicine, University of North Carolina at Chapel Hill, Chapel Hill, North Carolina, United States of America; Medical University Graz, AUSTRIA

## Abstract

**Background:**

In the US, medication assisted treatment, particularly with office-based buprenorphine, has been an important component of opioid dependence treatment among patients with iatrogenic addiction to opioid analgesics. The predictors of initiating buprenorphine for addiction among opioid analgesic patients have not been well-described.

**Methods:**

We conducted a time-to-event analysis using data from the North Carolina (NC) Prescription Drug Monitoring Program (PDMP). Our outcome of interest was time-to-initiation of sublingual buprenorphine. Our study population was a prospective cohort of all state residents receiving a full-agonist opioid analgesic between 2011 and 2015. Predictors of initiation of sublingual buprenorphine examined included: age, gender, cumulative pharmacies and prescribers utilized, cumulative opioid intensity (defined as cumulative opioid exposure divided by duration of opioid exposure), and benzodiazepine dispensing.

**Findings:**

Of 4.3 million patients receiving opioid analgesics in NC between 2011 and 2015 (accumulated 8.30 million person-years of follow-up), and a total of 28,904 patients initiated buprenorphine formulations intended for addiction treatment (overall rate 3.48 per 1,000 person-years). In adjusted multivariate models, the utilization of 3 or more pharmacies (HR: 2.93; 95% CI: 2.82, 3.05) or 6 or more controlled substance prescribers (HR: 12.09; 95% CI: 10.76, 13.57) was associated with buprenorphine initiation. A dose-response relationship was observed for cumulative opioid intensity (HR in highest decile relative to lowest decile: 5.05; 95% CI: 4.70, 5.42). Benzodiazepine dispensing was negatively associated with buprenorphine initiation (HR: 0.63; 95% CI: 0.61, 0.65).

**Conclusions:**

Opioid analgesic patients utilizing multiple prescribers or pharmacies are more likely to initiate sublingual buprenorphine. This finding suggests that patients with multiple healthcare interactions are more likely to be treated for high-risk opioid use, or may be more likely to be identified and treated for addiction. Future research should utilize prescription monitoring program data linked to electronic health records to include diagnosis information in analytic models.

## Introduction

Iatrogenic addiction to opioid analgesics has been prominently discussed as a driver of the overdose epidemic in the United States (US).[1] Patients who receive opioid analgesics for acute and chronic pain have a chance of developing addictive disorders involving this class of medications,[2] though the exact proportion who progress to addiction varies by pain treatment type, demographics, co-occurring mental health disorders, etc.[1, 3] Buprenorphine may also be prescribed to gradually taper and discontinue opioid analgesics in patients with chronic pain.[4] Sublingual buprenorphine is rarely prescribed for chronic or acute pain.[5] While not all patients with iatrogenic addiction to opioid analgesics receive office-based buprenorphine medication-assisted treatment (MAT) for addiction or tapering, the transition is of importance among physicians who treat pain in deciding on whether to obtain a buprenorphine waiver for MAT.[6] The transition is also important for evaluations of the adequacy of MAT coverage in the US and for estimating clinical resources needed for future treatment demand.[7–11]

In the ongoing opioid overdose epidemic in the United States, a leading demand-reduction paradigm suggests the need for abstinence from illicitly manufactured or diverted opioids. The medicalized treatment of opioid use disorder (OUD) is a major component of abstinence promotion. Medication assisted treatment (MAT) is a major OUD treatment modality, often characterized by the coupling of counseling and other mental health therapies and psychosocial supports with the use of an opioid agonist or antagonist. At present, there are three active pharmaceutical ingredients (APIs) used in MAT in the US: the antagonist naltrexone, and the agonists methadone and buprenorphine.

Buprenorphine-based MAT has been widely embraced,[12, 13] in part because of how it was regulated compared to methadone by the US Drug Addiction Treatment Act of 2000 (DATA 2000). Among its many advantages are its greater ease of access by rural patients, the possibility of prescribing by primary care providers, and reduced stigma for patients relative to methadone treatment program facilities.[14] Additionally, office-based buprenorphine can be an effective treatment in settings where medical management is available, but other behavioral or psychosocial components of MAT are either not available or desired.[15–18]

Currently, buprenorphine is widely available in outpatient settings and can be obtained for outpatient use from any physician who obtains a “buprenorphine waiver” and completes eight hours of training as required by DATA 2000. These trainings are administered annually nationwide by the Substance Abuse and Mental Health Services Administration (SAMHSA). DATA 2000 also established key regulations of buprenorphine MAT that make it unique among controlled substances (CS). Waivered physicians are limited in the maximum number of buprenorphine MAT patients they can serve at any one time. Newly-waived physicians also must complete a probationary period with even lower patient limits. In addition to maintenance dosing, waivered physicians may also use buprenorphine for shorter periods of opioid detoxification.

Buprenorphine is the only form of MAT prescribed by outpatient community physicians and dispensed by community pharmacies. As the only form of addiction treatment that is routinely captured in Prescription Drug Monitoring Program (PDMP) data, buprenorphine dispensing outcomes have been examined by others who have used these data.[19, 20] State-level PDMP data are generated electronically by specific pharmacies, primarily from outpatient and mail order dispensed prescriptions of controlled substances, and then uploaded to state government databases at regular time intervals as required. Unique patient, prescriber, and pharmacy identifiers are generated, and detailed drug identification is possible. These databases contain information on outpatient dispensed prescriptions for both opioid analgesics and on buprenorphine prescriptions by office-based MAT providers.

In this study, we used PDMP data to identify the predictors of transitions from opioid analgesic use (ostensibly for pain management) to outpatient sublingual buprenorphine use. We hypothesized that increasing cumulative exposure to opioids, increasing numbers of physicians and pharmacies utilized, male gender, younger age and benzodiazepine dispensing would increase the likelihood of buprenorphine use after prescribed opioid analgesic exposure. We were also interested in determining if there were thresholds or breakpoints in cumulative prescribed opioid analgesic exposure that may predispose a patient to subsequent MAT.

## Methods

### Setting and study overview

We conducted a retrospective cohort study of prospectively collected data among North Carolina (NC) residents, from January 1, 2011 to December 31, 2015, who received opioid analgesics (our exposure) to determine the predictors of progressing to outpatient sublingual buprenorphine use (our outcome), using time-to-event statistical models. Our sole data source was the state PDMP.

### Data

Records of all controlled substance prescriptions dispensed and recorded in the Controlled Substances Reporting System (CSRS, the NC PDMP) between January 1, 2011 and December 31, 2015 (60 months) were obtained from the NC Division of Mental Health, Developmental Disabilities, and Substance Abuse Services (DMHDDSAS). Cohort members were tracked longitudinally via unique identifiers created by the data vendor (Appriss, Louisville, KY) using a proprietary method based on their names, dates of birth, and addresses. Unique prescriber and pharmacy identifiers were derived from Drug Enforcement Administration (DEA) license numbers.

Relevant controlled substances dispensed were identified using National Drug Code (NDC) numbers. Product dosage and formulation details were abstracted using natural language processing and merged with drug metadata previously collected by our team: the active pharmaceutical ingredient (API), class (e.g., immediate-release full mu-opioid receptor agonist, benzodiazepine, stimulant), formulation (e.g., tablet, sublingual film, liquid), and dose.[21, 22] Oral morphine equivalents were calculated for oral and transdermal full mu-opioid receptor agonists, excluding liquid opioid preparations used primarily as cough suppressants.

### Cohort and outcome definition

We constructed a cohort of prescription opioid analgesic (OA) recipients residing in North Carolina using the 2011–2015 PDMP data. To be eligible for cohort retention, NC residents had to receive at least one additional CS after their first dispensed OA, to reduce unobservable time bias. Cohort members were considered outcome-positive at the time of their first dispensed prescription for MAT-indicated formulations of buprenorphine (e.g., Suboxone, Subutex, and generics), at which point follow-up stopped. Outcome-negative cohort members were censored at the time of their last filled CS.

Additional covariates captured through the PDMP were cohort members’ age, sex, cumulative pharmacies and prescribers utilized, cumulative opioid intensity (defined as cumulative opioid exposure divided by duration of opioid exposure), and dispensing of benzodiazepines. Age was captured as an 11-level categorical variable, using ages 40–44 as the referent category, traditionally an age group with the highest overdose mortality. Sex and benzodiazepine exposure were modeled as binary, non-time-varying covariates. Cohort members with any history of benzodiazepine dispensing at cohort entry or during follow-up were considered exposed to benzodiazepines. Cumulative totals of pharmacies and prescribers used to-date by cohort members for any CS were captured as time-varying 3- and 6-level categorical variables, respectively, based on empirical examination of probability distributions.

### MME calculation

In order to assess whether total exposure to opioid analgesics may influence subsequent buprenorphine MAT, cumulative morphine milligram equivalents (MMEs) per prescription were calculated by multiplying the number of units dispensed by the days supply by a conversion factor.[21, 23] Cumulative MMEs over time for each patient were then calculated by adding MMEs from each prescription dispensed during the observation period. For each individual, the cumulative MMEs dispensed were divided by the cohort member’s person-time at-risk, creating a time-varying index of the current OA exposure density, interpreted as average daily MME, taking into account overlapping prescriptions. A 10-level categorical treatment of this measure was created based on rate deciles.

### Statistical methods

**Fig 1:**
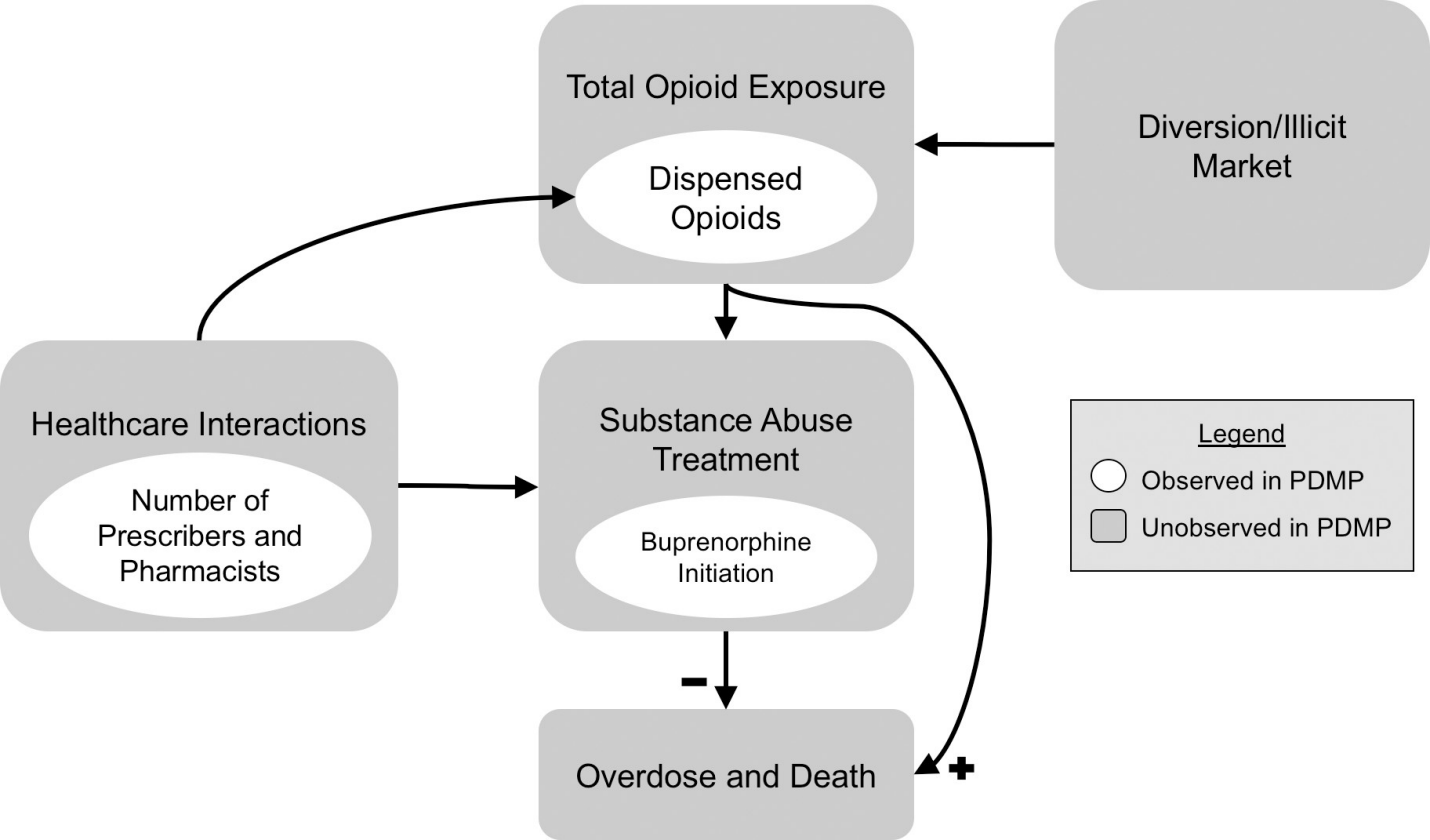
Abbreviated directed acyclic graph of buprenorphine initiation and prescription drug monitoring program data elements. Elements shown in gray squares cannot be directly observed in Prescription Drug Monitoring (PDMP) data, while elements in white ovals can be observed.

Parameterization and the proportional hazards assumptions of the above measures were assessed empirically using frequency distributions and histograms, Kaplan-Meier survival plots, Nelson-Aalen cumulative hazard plots, and log of negative-log survival plots. We estimated hazard ratios using multivariate Cox proportional hazards regression, with robust standard errors to account for multiple observations per cohort member. Interactions with time-varying benzodiazepine exposure and cumulative MME exposure were also assessed, based on a directed acyclic graph (DAG) model of buprenorphine initiation and iatrogenic addiction (Fig 1). The 2011–2015 period of the analysis was likely to be influenced by underlying secular trends in opioid use (including buprenorphine) and overdose, changes in clinical practice, and other interventions administered.[21] To account for secular trends, we adjusted for calendar time as continuous month over the 60-month observation period.

To improve computational efficiency, regression models were estimated on a randomly sampled dataset containing 10% of all censored opioid analgesic patients and 100% of patients initiating buprenorphine. Thus, our regression models used all outcome-positive patients and a 10% random sample of outcome-negative patients, where the outcome was defined as initiating buprenorphine. We created sample weights based on the inverse probability of sampling (i.e., 1 for outcome-positive or 10 for censored), and used survey analysis methods to ensure that our point estimates and confidence intervals were statistically correct. This approach was validated against univariate models of the binary predictors that used the entire (i.e., unsampled) dataset of 54,594,418 records. All population-based epidemiologic measures (such as the size of population studied and the incidence rate for initiating buprenorphine) were computed using the entire dataset.

Analyses were conducted in a distributed server environment using Stata/MP 14.1 and Stata/IC 15.0 (StataCorp, College Station, TX).

### Ethics review

This study was approved by the institutional review board of the University of North Carolina at Chapel Hill (IRB 17–0889).

## Results

### Descriptive findings

**Fig 2:**
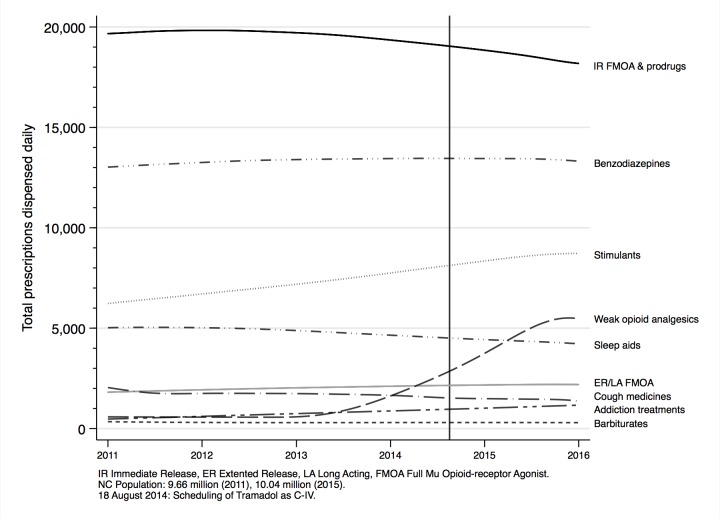
Daily prescribing of controlled substances by class, North Carolina, 2011-2015. During the study period, immediate release full mu opioid receptor agonist and agonist prodrug analgesics accounted for the most controlled substance prescriptions daily. The class of controlled substances with the greatest absolute and relative change was the weak opioid analgesic class. This was due to the scheduling of tramadol as a C-IV controlled substance during the study period.

**Fig 3:**
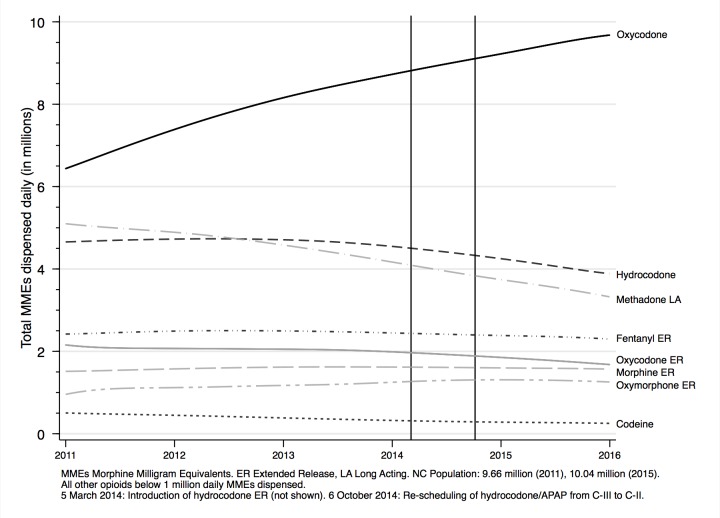
Oral full mu-agonist opioid analgesic and transdermal fentanyl dispensing by active pharmaceutical ingredient, North Carolina, 2011–2015. Notable market regulatory changes to full mu-agonists during the study period included the introduction of hydrocodone extended-release (ER), and the rescheduling of hydrocodone/acetaminophen from a C-III to C-II controlled substance. Hydrocodone ER dispensing was very low and would not be interpretable on the scale of this figure.

Trends of CS and OA dispensing in North Carolina are shown in Fig 2 and Fig 3. During the study period (January 2011 –December 2015), dispensing of prescriptions for immediate release (IR) opioids decreased gradually, whereas stimulant dispensing increased. Tramadol, a weak opioid, was first scheduled as a controlled substance nationwide in 2013, and was not consistently reported prior to then. Dispensing of other classes of controlled substances, including extended release (ER) OAs, did not change dramatically. However, the total volume of opioids dispensed increased markedly during this time period. Outpatient addiction treatment prescriptions, namely buprenorphine MAT, experienced linear growth over the study period, though initial prescribing was low relative to other CS. Specifically, monthly dispensing increased from around 15,000 scripts at the beginning of the study period to around 35,000 at the end of the study period, with a steady monthly increase of 347 scripts each month (95% CI: 332, 362).

Among the full agonist opioids (Fig 3), MMEs of oxycodone IR dispensed increased over 38 percent, while hydrocodone IR and methadone Long Acting (LA) dispensing decreased, although to a lesser extent. While the most-prescribed OA APIs were hydrocodone, oxycodone, codeine, and morphine, the most-dispensed by MMEs were oxycodone, hydrocodone, methadone, and fentanyl. The decline in hydrocodone IR dispensing began before combination products were further restricted by rescheduling in mid-2014; hydrocodone ER was too minimal to account for the decline in IR formulations.

### Time-to-event analysis of buprenorphine initiation

Approximately 4.3 million patients were included in the cohort of opioid analgesic recipients contributing 8.30 million person-years of follow-up during the 2011–2015 period, of which 28,904 initiated buprenorphine (with an overall rate of 3.48 per 1,000 person-years among opioid analgesic patients). Our sampled analysis dataset included a 10% sample of 432,594 censored patients and all patients who initiated buprenorphine (Table 1).

**Table 1:** Time-to-event analysis cohort of opioid analgesic patients in North Carolina, 2011–2015.

	**OA patients receiving buprenorphine**								
**Measure**	**Mean (n)**	**SD (%)**	**Minimum**	**p10**	**p25**	**Median**	**p75**	**p90**	**Maximum**
*n*	28904								
Males (n, %)	13949	48							
Co-prescribed benzodiazepine (n, %)	12694	43.9							
Age at cohort entry	34.62	11.29							
Person-days at risk	619.77	475.1	1	62	199	525	973	1336	1817
Total Pharmacies Utilized	3.51	2.81	1	1	2	3	4	7	37
Total Prescribers Utilized	6.26	5.55	1	2	3	4	8	13	87
Opioid Analgesic scripts filled while at risk	12.96	19.71	1	1	2	5	15	37	386
Cumulative MMEs Dispensed while at risk	24252	88961	0	100	250	1241	10278	53265	3559633
	**OA patients not receiving buprenorphine**								
	**Mean (n)**	**SD (%)**	**Minimum**	**p10**	**p25**	**Median**	**p75**	**p90**	**Maximum**
*n*	4313402								
Males (n, %)	1830471	42.4							
Co-prescribed benzodiazepine (n, %)	1541637	35.7							
Age at cohort entry	47.71	18.7							
Person-days at risk	697.9	564.83	1	31	176	586	1160	1577	1825
Total Pharmacies Utilized	2.03	1.53	1	1	1	2	2	4	79
Total Prescribers Utilized	4.03	3.32	1	2	2	3	5	8	159
Opioid Analgesic scripts filled while at risk	8.26	15.63	1	1	2	3	6	20	651
Cumulative MMEs Dispensed while at risk	10353	56308	0	150	255	600	2450	14238	6462773

MME Milligram Morphine Equivalent, MAT Medication Assisted Treatment

Gender was similar among those who initiated sublingual buprenorphine (48% male) compared to those who were censored (43% male). The mean age at cohort entry was lower among the buprenorphine initiators (34.6 years, SD: 11.3) than among censored patients (47.7 years, SD: 18.7). Controlled substance dispensing differed between the groups, insofar as outcome-positive sublingual buprenorphine initiators were exposed to considerably more opioid substances through prescriptions. Buprenorphine-initiating patients filled an average of 13.0 opioid analgesic prescriptions (median: 5), while their censored counterparts filled an average of 8.3 (median: 3). Additionally, buprenorphine initiators contributed an average 620 days of follow-up (SD: 475), 78 fewer days of follow-up compared to non-initiators (mean: 697, SD: 565). Furthermore, mean cumulative opioid analgesic exposure was more than twice as high among sublingual buprenorphine initiators: 24,252 MMEs (median: 1,241) versus 10,353 MME (median: 600).

In unadjusted Cox models of buprenorphine initiation, statistically significant associations were observed for all predictors (Table 2). Male gender was associated with a 40-percent increase in the Hazard Ratio of buprenorphine initiation (HR: 1.40; 95% CI: 1.36, 1.43). The HRs of buprenorphine initiation were notably higher among 20-34-year-olds compared to the 40–44 population typically experiencing the highest OA overdose mortality. Adults over 60 also had very low HRs, as low as 0.05 among those 65 and older (95% CI: 0.05, 0.06). Three or more total pharmacies compared to one (HR: 6.97; 95% CI: 6.73, 7.21) and 6 or more total prescribers (HR: 26.01; 95% CI: 23.29, 29.05) utilized compared to one were very strongly associated with increased rates of buprenorphine initiation. These effects were also notably higher in magnitude than measures of cumulative OA exposure, though increasing OA exposure was also strongly associated with buprenorphine initiation, and a clear dose-response was observed. Patients ever dispensed benzodiazepines were associated with a decreased hazard of buprenorphine initiation (HR: 0.91; 95% CI: 0.89, 0.93).

**Table 2:** Proportional hazards regression of first buprenorphine initiation among North Carolina opioid analgesic recipients, 2011–2015.

				Unadjusted Univariate Models			Calendar-time Adjusted		
							Multivariate Model		
Measure		Number of Events	Person-years at risk	HR	95% CI	CLR	HR	95% CI	CLR
**Gender**									
	Female	14,853	4,947,296	1 (Ref.)			1 (Ref.)		
	Male	13,944	3,340,178	1.4	1.36, 1.43	1.05	1.5	1.46, 1.54	1.06
**Age**									
	0–19	587	366,872	0.44	0.40, 0.48	1.2	0.58	0.53, 0.64	1.211
	20–24	4,063	389,322	2.83	2.69, 2.98	1.109	3.9	3.68, 4.13	1.121
	25–29	5,846	466,256	3.35	3.20, 3.52	1.102	4.02	3.81, 4.24	1.114
	30–34	5,401	555,510	2.57	2.45, 2.70	1.102	2.81	2.67, 2.97	1.113
	35–39	3,943	608,898	1.68	1.59, 1.76	1.107	1.76	1.66, 1.85	1.115
	40–44	2,835	726,626	1 (Ref.)			1 (Ref.)		
	45–49	2,159	783,025	0.7	0.66, 0.74	1.123	0.67	0.63, 0.71	1.129
	50–54	1,850	886,790	0.52	0.49, 0.55	1.129	0.49	0.46, 0.52	1.137
	55–59	1,275	857,495	0.36	0.34, 0.39	1.146	0.35	0.33, 0.38	1.153
	60–64	534	756,742	0.17	0.16, 0.19	1.207	0.18	0.16, 0.20	1.213
	65+	404	1,901,437	0.05	0.05, 0.06	1.234	0.07	0.06, 0.07	1.239
**Co-prescribed Benzodiazepine**									
	None	16,210	4,514,716	1 (Ref.)			1 (Ref.)		
	Any	12,694	3,783,710	0.91	0.89, 0.93	1.05	0.63	0.61, 0.65	1.065
**Total Pharmacies Utilized**									
	1	5,316	3,745,380	1 (Ref.)			1 (Ref.)		
	2	8,511	2,767,967	2.31	2.23, 2.23	1	1.79	1.72, 1.85	1.076
	3 or more	15,077	1,786,995	6.97	6.73, 7.21	1.071	2.93	2.82, 3.05	1.083
**Total Prescribers Utilized**									
	1	340	652,977	1 (Ref.)			1 (Ref.)		
	2	6,143	2,737,029	4.74	4.24, 5.28	1.245	5.13	4.59, 5.75	1.252
	3	4,664	1,759,069	6.57	5.88, 7.34	1.248	5.98	5.34, 6.71	1.256
	4	3,515	1,079,261	8.9	7.96, 9.96	1.252	7.06	6.29, 7.93	1.262
	5	2,805	676,523	12.1	10.79, 13.56	1.257	8.35	7.42, 9.41	1.268
	6 or more	11,437	1,394,387	26.01	23.29, 29.05	1.248	12.09	10.76, 13.57	1.261
**Decile of cumulative MME Exposure**									
**(Cum. MMEs / Cum. days exposed)**									
	[0, 0.44)	4,480	2,307,461	1 (Ref.)			1 (Ref.)		
	[0.44, 1.05)	3,919	1,741,273	1.17	1.12, 1.23	1.091	0.94	0.90, 0.98	1.097
	[1.05, 2.33)	3,746	1,201,369	1.62	1.55, 1.69	1.093	1.25	1.19, 1.31	1.103
	[2.33, 5.00)	3,124	834,771	1.93	1.84, 2.02	1.098	1.6	1.52, 1.69	1.112
	[5.00, 9.95)	2,640	583,162	2.31	2.20, 2.43	1.104	2.07	1.95, 2.19	1.123
	[9.95, 17.76)	2,154	430,116	2.53	2.40, 2.67	1.112	2.45	2.30, 2.61	1.133
	[17.76, 30.00)	1,962	355,099	2.77	2.62, 2.93	1.118	2.73	2.56, 2.92	1.141
	[30.00, 50.76)	1,949	312,936	3.12	2.95, 3.30	1.119	3.13	2.93, 3.35	1.145
	[50.76, 112.69)	2,282	280,356	4.05	3.84, 4.28	1.115	3.75	3.51, 4.02	1.146
** **	[112.69, 60400)	2,648	252,570	5.22	4.94, 5.50	1.113	5.05	4.70, 5.42	1.152

HR Hazard Ratio, CI Confidence Interval, CLR Confidence Limit Ratio, MME Morphine Milligram Equivalents

**Fig 4:**
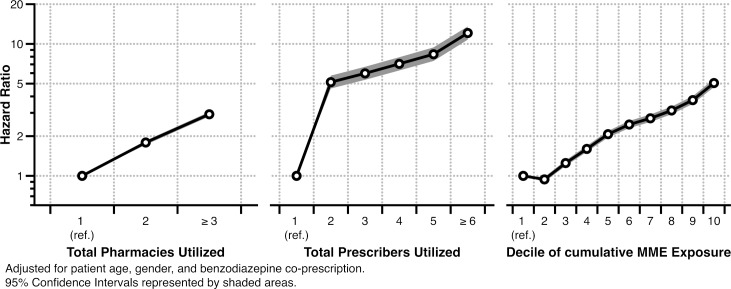
Multivariate adjusted Hazard Ratios of first buprenorphine initiation among North Carolina opioid analgesic recipients, 2011-2015. Note that the Hazard Ratios are plotted on a logarithmic scale.

In calendar-time adjusted, multivariable models, these associations persisted. Younger male OA patients had higher hazard ratios of buprenorphine initiation, particularly ages 20–34 (HR in 25-29-year-olds: 4.02; 95% CI: 3.81, 4.24). The negative association between dispensed benzodiazepines and buprenorphine initiation increased in magnitude (HR: 0.63, 95% CI: 0.61, 0.65). The effects of total pharmacies and prescribers were somewhat attenuated. Patients using 3 or more pharmacies to fill controlled substances (HR: 2.93; 95% CI: 2.82, 3.05), and patients with 6 or more controlled substance prescribers utilized (HR: 12.09; 95% CI: 10.76, 13.57) remained very strongly associated with buprenorphine initiation. Patients in the 20^th^-and-higher-percentile of cumulative opioid exposure experienced a steadily increasing hazard of buprenorphine initiation up to 4 times higher in the highest decile than patients in the lowest decile (HR: 5.05; 95% CI: 4.70, 5.42). The associations observed in the multivariable adjusted model for these three measures are also graphically demonstrated in Fig 4.

## Discussion

As Prescription Drug Monitoring Programs have been widely promoted as an important supply-reduction tool, we also sought to assess their potential utility for demand-reduction: that is, by identifying patients filling prescriptions for opioid analgesics with a high likelihood of initiating MAT with buprenorphine. Buprenorphine initiation itself must be characterized as a key event in the health trajectory of a patient with OUD. Whether intended for MAT, detoxification, or tapering, the formulations of buprenorphine used as our outcome are all forms of demand-reduction that address the addictive potential of OAs while continuing to provide effective analgesia. Therefore, while buprenorphine initiation constitutes a potentially positive event in a patient’s history, it is also a possible marker of the need for improved pain and opioid management.

It is generally accepted that outpatient exposure to opioid analgesics can lead to iatrogenic addiction, which in turn can be treated with MAT, including (but not limited to) sublingual buprenorphine. While the intermediate outcome of iatrogenic addiction itself is not readily observable in large population-based data sources, we were able to observe the subsequent transition to buprenorphine, which is a key factor in clinical resource allocation in the opioid crisis in the US. Not surprisingly, on an individual level, cumulative exposure to opioids was associated with increased likelihood of buprenorphine dispensing, ostensibly for MAT, with a near-monotonic dose-response relationship. What was surprising, however, was that the likelihood of receiving sublingual buprenorphine was elevated at doses well below the clinical threshold of 90 MME/day used in the Centers for Disease Control and Prevention (CDC) guideline for preventing overdose, with HR 3.75 (95% CI: 3.51, 4.02) for 51-to-113 cumulative daily MME.[24] This observation is consistent with a recent review by the US Food and Drug Administration that found opioid analgesics to have risks at all doses, not just those above arbitrary thresholds.[25]

Our finding that buprenorphine initiation was notably less likely among OA patients who were ever dispensed benzodiazepines was also unexpected. A previous study found that 1 in 8 buprenorphine MAT prescriptions were used concomitantly with benzodiazepines.[20] Since the publication of the CDC guideline and the institution of boxed warnings on opioids and benzodiazepines,[24] overdose prevention efforts have increasingly sought MME reduction as a first-line supply-side intervention, along with stricter controls of co-prescriptions of OA and benzodiazepines.[26] Unfortunately, our data cannot elucidate why benzodiazepine exposure may be protective of buprenorphine initiation. Benzodiazepines may be considered an informal clinical contraindication for buprenorphine, or they may suggest comorbidity between pain and anxiety or sleep disorders for which benzodiazepines are routinely prescribed, or some other unobserved factor. It also may be an indicator of patient characteristics associated with low coordination between multiple providers. In addition, this association may be confounded by the association between co-prescribing and fatal overdose. The rate of certain events that would result in right-censoring, such as fatal overdose, or possibly entry into residential treatment, are higher in patients utilizing both OA and benzodiazepines.[22]

OA patients with increasing numbers of CS-prescribing physicians, as well as increasing numbers of pharmacies used, had a greatly increased rate of initiating buprenorphine, even after for controlling for the intensity of cumulative opioids. The number of providers-to-date in particular constitutes a proxy measure of the total volume of healthcare interactions patients have. It is possible that patients with a high volume of healthcare interactions are good candidates for outpatient MAT with buprenorphine, or initiate buprenorphine to address high-risk opioid use because of the coordination of multiple prescribers.

The simplified directed acyclic graph (DAG) model of the PDMP data utilized (Fig 1) suggests some possible explanations for our results. The measures captured through a PDMP, such as dispensed opioids, the number of pharmacists and prescribers utilized, and use of buprenorphine, are only a few components of patients’ true exposures, and subject to distinct bias. Patients with the greatest risk of substance abuse or overdose also have the poorest estimates of total opioid exposure, because of their use of shared prescription opioids, and, increasingly, the illicit opioid market.[27–29] Our study was also limited to buprenorphine dispensed on an outpatient basis, as other forms of substance abuse treatment, including MAT with methadone and naltrexone, inpatient residential, and counseling-only modalities, are not captured through PDMPs. Finally, measures that can be derived from the number and identity of prescribers and pharmacists utilized are only proxies of healthcare interactions.

Though these proxies may have utility for purposes such as law enforcement, they do not reflect key risk and protective factors such as nonfatal overdose, inpatient opioid exposure, quality and integration of specialist care received (including pain management), and insurance status. The gap between data elements observed and unobserved in PDMP data can be bridged through the linkage of PDMP data with electronic health records, particularly ED data concerning nonfatal overdose, and treatment program quality assurance data. A more complete estimation of the quality of healthcare interactions would also allow population-level evaluation of medical home approaches to pain management. Linkage of this nature may allow clinicians and researchers to better address the biases present in this model of OA use and MAT. However, these linkages naturally raise many challenging data privacy questions.

Our study was conducted using PDMP data from 2011 through 2015, and the results should be interpreted in light of the history of that period in the US. The “triple wave” framework of prescription, heroin, and fentanyl overdose epidemics is a useful model for considering this period.[30] 2011 is considered a peak year in the first wave marked by high prescription opioid prescribing, and the year that heroin overdose deaths (a second wave marker) began to markedly increase. 2015 is noteworthy as the year that heroin overdose deaths overtook deaths attributed to prescription opioids. The third wave of synthetic opioids, including illicitly manufactured fentanyl, is also first noted during our study period, with the dramatic increasing in synthetic opioid deaths first observed in 2013. These national trends are similar to those observed in NC, where gradual decreases in prescription opioid overdose deaths occurred alongside substantial increases in heroin and synthetic opioid deaths.[31]

### Limitations

The use of unlinked PDMP data for a survival analysis presents certain limitations related to the measurement of time-at-risk. After the dispensing of the first recorded OA, patients can only contribute time at-risk as long as they continue to fill CS prescriptions in North Carolina. Therefore, a patient would have to fill at least two prescriptions for a CS during this time period, the first of which was for an OA. Given that competing risks included death (including overdose), leaving NC, and entry into other forms of addiction treatment or MAT, we considered this conservative approach superior to assuming that recipients of a single OA were still alive at the end of the follow up period. Strong negative associations observed in older patients may be attributable to competing risks not detectable in the PDMP, including death, hospitalization or inpatient addiction treatment, underutilization of MAT in this population, or the use of transdermal buprenorphine as an alternative pain management approach. Many of these concerns can be directly addressed in future studies via PDMP linkage as described above.

Days supply, which was used to calculate average MME, is a field that is created by the pharmacist at the point of dispensing and has not been validated. Our models necessarily assumed that opioid analgesics and buprenorphine were taken as prescribed; ingestion of diverted opioids (e.g., from sources not captured in the PDMP such as sharing or street purchases) was not observable in the data source. The PDMP thus may undercount opioid analgesic exposure for those using other opioids, or over-represent exposure if the prescriptions were diverted to others. Use of diverted buprenorphine products is itself a complex phenomenon, often with motivations related to withdrawal self-management and self-detoxification, outside the scope of our analyses of a patient’s first dispensed buprenorphine prescription.[32]Without mandated standard practices, the quality of PDMP data varies greatly between states. In NC, metrics of the quality of entity resolution for patients, as well as prescribers and pharmacies, have been poorly described. Our dataset included up to three additional associated prescriber IDs per prescription which were used in conjunction with the primary ID to create a single synthetic unified ID. Our patient IDs were based on a vendor-developed proprietary algorithm using name, date of birth, and address for entity resolution. However, no published documentation of the quality of patient resolution algorithms was available for the NC PDMP. It is entirely possible that patients without stable medical homes might also lack stable homes of their own, and accumulate multiple un-linked IDs. The NC PDMP began full data collection in 2009; apart from the aforementioned issues with patient identification it is unlikely that controlled substance prescriptions were not captured.

Our findings and conclusions must also be considered in light of the undertreatment of opioid use disorder during the study period. Older patients who received opioid analgesics, in particular, had very low hazard ratios of buprenorphine initiation. MAT remains underutilized relative to the total population of individuals with opioid use disorder.

## Conclusion

In prescription drug monitoring data, opioid analgesic patients utilizing multiple prescribers or pharmacies were more likely to initiate buprenorphine. Patients with multiple healthcare interactions may be more likely to have high-risk opioid use, including dependence, identified and treated on an outpatient basis. More intense cumulative opioid exposure also increased the rate of buprenorphine initiation. The dispensing of benzodiazepines to opioid analgesic patients may be an indicator of patient characteristics associated with low coordination among multiple providers. Increased specificity of these metrics and other reductions in bias will be possible through linkage of PDMP data with electronic health records.
